# A stakeholder-driven method for selecting implementation strategies: a case example of pediatric hypertension clinical practice guideline implementation

**DOI:** 10.1186/s43058-022-00276-4

**Published:** 2022-03-07

**Authors:** Ashley A. Knapp, Allison J. Carroll, Nivedita Mohanty, Emily Fu, Byron J. Powell, Alison Hamilton, Nicole D. Burton, Elaine Coldren, Tania Hossain, Dhanya P. Limaye, Daniel Mendoza, Michael Sethi, Roxane Padilla, Heather E. Price, Juan A. Villamar, Neil Jordan, Craig B. Langman, Justin D. Smith

**Affiliations:** 1 Department of Psychiatry and Behavioral Sciences, Northwestern University Feinberg School of Medicine, Chicago, IL USA; 2 Department of Psychiatry and Behavioral Sciences and Preventive Medicine, Northwestern University Feinberg School of Medicine, Chicago, IL USA; 3 Alliance Chicago, Chicago, IL USA; 4 Center for Mental Health Services Research, Brown School & School of Medicine, Washington University in St. Louis, St. Louis, MO USA; 5 VA Center for the Study of Healthcare Innovation, Implementation & Policy, VA Greater Los Angeles Healthcare System, Los Angeles, CA USA; 6 Department of Psychiatry and Biobehavioral Sciences, David Geffen School of Medicine, University of California Los Angeles, Los Angeles, CA USA; 7 Near North Health, Chicago, IL USA; 8 Erie Family Health Centers, Chicago, IL USA; 9 Heartland Health Centers, Chicago, IL USA; 10 Lawndale Christian Health Center, Chicago, IL USA; 11 Stanley Manne Children’s Research Institute, Ann & Robert H. Lurie Children’s Hospital of Chicago and Department of Pediatrics, Northwestern University Feinberg School of Medicine, Chicago, IL USA; 12 Center of Innovation for Complex Chronic Healthcare, Hines VA Hospital, Hines, IL USA; 13 Ann & Robert H. Lurie Children’s Hospital of Chicago and Department of Pediatrics, Northwestern University Feinberg School of Medicine, Chicago, IL USA; 14 Department of Population Health Sciences, Spencer Fox Eccles School of Medicine at the University of Utah, Salt Lake City, UT USA

**Keywords:** Expert Recommendations for Implementing Change, Implementation Research Logic Model, User-centered design, Implementation strategy, Pediatric hypertension, Stakeholder engagement

## Abstract

**Background:**

This article provides a generalizable method, rooted in co-design and stakeholder engagement, to identify, specify, and prioritize implementation strategies. To illustrate this method, we present a case example focused on identifying strategies to promote pediatric hypertension (pHTN) Clinical Practice Guideline (CPG) implementation in community health center-based primary care practices that involved meaningful engagement of pediatric clinicians, clinic staff, and patients/caregivers. This example was chosen based on the difficulty clinicians and organizations experience in implementing the pHTN CPG, as evidenced by low rates of guideline-adherent pHTN diagnosis and treatment.

**Methods:**

We convened a Stakeholder Advisory Panel (SAP), comprising 6 pediatricians and 5 academic partners, for 8 meetings (~12 h total) to rigorously identify determinants of pHTN CPG adherence and to ultimately develop a testable multilevel, multicomponent implementation strategy. Our approach expanded upon the Expert Recommendations for Implementation Change (ERIC) protocol by incorporating a modified Delphi approach, user-centered design methods, and the Implementation Research Logic Model (IRLM). At the recommendation of our SAP, we gathered further input from youth with or at-risk for pHTN and their caregivers, as well as clinic staff who would be responsible for carrying out facets of the implementation strategy.

**Results:**

First, the SAP identified 17 determinants, and 18 discrete strategies were prioritized for inclusion. The strategies primarily targeted determinants in the domains of intervention characteristics, inner setting, and characteristics of the implementers. Based on SAP ratings of strategy effectiveness, feasibility, and priority, three tiers of strategies emerged, with 7 strategies comprising the top tier implementation strategy package. Next, input from caregivers and clinic staff confirmed the feasibility and acceptability of the implementation strategies and provided further detail in the definition and specification of those strategies.

**Conclusions:**

This method—an adaptation of the ERIC protocol—provided a pragmatic structure to work with stakeholders to efficiently identify implementation strategies, particularly when supplemented with user-centered design activities and the intuitive organizing framework of the IRLM. This generalizable method can help researchers identify and prioritize strategies that align with the implementation context with an increased likelihood of adoption and sustained use.

**Supplementary Information:**

The online version contains supplementary material available at 10.1186/s43058-022-00276-4.

Contributions to the literature

- This study presents a rigorous and replicable process for meaningfully engaging stakeholders and implementation partners in the selection, specification, and prioritization of implementation strategies.
- This process augments the Expert Recommendation for Implementing Change (ERIC) protocol with user-centered design activities and the Implementation Research Logic Model (IRLM).
- To exemplify this method, we present a case example in which stakeholders guided the identification of a multilevel, multicomponent implementation strategy for CPG implementation for the diagnosis and management of pHTN.
- The methodology described in this article can be applied to improve the likelihood of strategy effectiveness and sustainment for a variety of implementation projects.

## Background

A ubiquitous challenge for implementation researchers is selecting appropriate implementation strategies to improve the adoption, implementation, and sustainment of effective interventions. Oftentimes, implementation strategies are selected based on theory and prior research. For example, implementation researchers may use the Expert Recommendations for Implementing Change (ERIC) [1], a compilation of 73 discrete implementation strategies that was developed through a modified Delphi process with a wide range of stakeholders and is useful for identifying strategies and matching them to identified determinants [2]. Powell et al. [3] have also proposed a variety of methods for matching implementation strategies to identified barriers and facilitators, including concept mapping, group model building, conjoint analysis, and intervention mapping. However, following theoretical methods or relying solely on prior research does not always successfully translate to a new context and/or for a particular intervention as these methods do not account for on-the-ground stakeholder knowledge and preferences.

To overcome limitations of other methods for selecting implementation strategies, we propose a rigorous and generalizable stakeholder-driven method. Stakeholder engagement is a keystone of implementation research [4]. Stakeholders are often engaged throughout the research process; however, they are most likely to be engaged in data synthesis and dissemination (i.e., later in the implementation process) [5]. Engaging stakeholders in the identification, operationalization, and selection of implementation strategies is more likely to produce strategies that will be taken up and result in adoption and sustained implementation.

Herein, we illustrate a rigorous stakeholder-driven method for selecting implementation strategies using a case example with the goal of developing a multilevel, multicomponent strategy for the implementation of the clinical practice guidelines (CPG) for pediatric hypertension (pHTN) in safety-net community health centers [6].

### Case example: pediatric hypertension clinical practice guideline implementation

Despite CPGs for pHTN being in place for decades [7, 8], evidence indicates poor adherence [7, 9–11]. Between 2 and 4% of children in the US general population have pHTN [12], and over 16% have elevated BP (previously called pre-hypertension) [13, 14]. In one electronic health record (EHR) review, nearly 85% of children who met the criteria for elevated blood pressure (BP) or pHTN were undiagnosed [15]. Guideline-adherent pHTN diagnoses are highly predictive of having HTN as an adult [16]. The consequences of untreated pHTN include left ventricular hypertrophy, neurocognitive deficits, and target organ damage in adolescence [17–19], as well as hypertension, metabolic syndrome, and left ventricular hypertrophy in adulthood [16, 20].

Numerous barriers to CPG adherence have been identified [11, 21]. One qualitative study found that primary care clinicians perceived significant barriers at both the system and patient levels. These included lack of systematic approach to measuring (e.g., children not sitting still; lack of proper equipment; not using manual BP readings) and reviewing BP values, difficulty interpreting BP readings and coordinating reassessment and necessary clinical actions within the workflow, and difficulty scheduling and completing follow-up appointments [22]. Parents of children with pHTN have also expressed uncertainty about diagnostic accuracy and treatment indication [23].

Identifying and testing implementation strategies to overcome existing barriers and improve pHTN CPG adherence is needed to prevent chronic illness. For example, clinical decision support (CDS) tools within the EHR have been shown to increase the detection and control of hypertension in adults [24, 25]. In contrast, pediatric clinicians using an EHR with such CDS failed to diagnose an alarming 95% of 3- to 17-year-olds whose BP measurements indicated meeting diagnostic criteria for pHTN per the 2017 CPG [26]. The fact that many children and adults with hypertension remain undetected [10] demonstrates that, although promising, health information technologies (HIT) used in isolation (i.e., without strategies to support their use and other aspects of the CPG) may be insufficient for guideline-adherent diagnosis and management [27].

The challenge of CPG adherence is not unique to pHTN. In a scoping review of barriers to CPG adherence and the strategies to overcome them, Fischer et al. [28] broadly grouped implementation strategies into workflow- or clinician-focused. Workflow-focused strategies included CDS tools, as well as standardized documentation and standing orders. Clinician-focused strategies largely focused on communication strategies (e.g., educational materials, ongoing trainings, social interactions between clinicians and opinion leaders). Fischer et al. further noted the importance of tailoring these strategies for the specific condition and setting. A similar process of identifying a multilevel, multicomponent strategy has not been undertaken specifically for pHTN CPG adherence. pHTN CPG adherence was chosen as an exemplar case given the low rates of guideline-adherent pHTN diagnosis and treatment, underscoring the difficulty of clinicians' and organizations' experience in implementing strategies to support adherence to pHTN CPG. The final implementation strategy will be multilevel, as patients, clincians, leadership, and policymakers influence pHTN CPG implementation.

### Present study

This article presents a generalizable method focused on meaningful engagement with stakeholders in the identification, specification, and prioritization of implementation strategies. To identify strategies tailored for the condition and setting, as recommended by Fischer et al. [28], we expanded upon an adapted Expert Recommendations for Implementation Change (ERIC) protocol [1, 29, 30]. Specifically, we used a modified Delphi approach, user-centered design activities [31], and the Implementation Research Logic Model (IRLM) [32] in a series of iterative meetings with stakeholders. Through the case example, we illustrate the steps involved in this method for implementation strategy identification, specification, and prioritization and then discuss the advantages of using this process, alternative methodologic considerations, and implications for implementation science.

## Methods

### Participants

#### Academic-community partnership

This study is grounded in an academic-community partnership that is the result of longstanding collaborations between Northwestern University Feinberg School of Medicine, Lurie Children’s Hospital of Chicago, and AllianceChicago, an AHRQ-recognized Practice Based Research Network comprising 60 community health centers with more than 200 clinic sites in 19 states as of 2021.

#### Stakeholder Advisory Panel (SAP)

The SAP comprised pediatric healthcare clinicians and research team members. Pediatric clinicians (*n*=6) were pediatric or family medicine physicians that would represent the perspective of community health center-based primary care practices that intended to participate in a subsequent implementation trial. The research team members (*n*=5) who led and participated in the meetings had expertise in pHTN diagnosis and treatment, implementation science focused on chronic disease management, user-centered design, pediatric primary care, health disparities, and use of HIT to support CPG adherence. Pediatric clinicians were recruited from four community health center organizations in the Chicago area that routinely collaborate in practice transformation initiatives using AllianceChicago’s HIT and practice change infrastructure.

### Procedures and case example

**Fig. 1:**
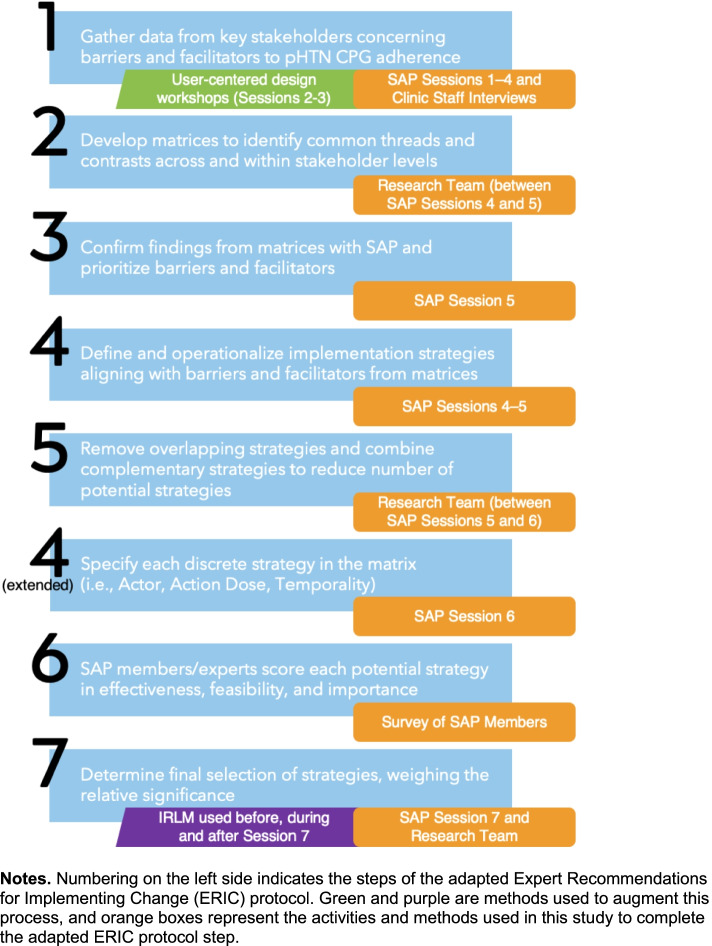
Adapted ERIC protocol with user-centered design and IRLM augmentations

We used a pragmatic adaptation and expansion of the ERIC protocol (see Fig. 1 for alignment of study activities with the steps of the ERIC protocol) to engage stakeholders in identifying, specifying, and prioritizing implementation strategies [1, 29, 30]. ERIC involves an iterative, multi-method process of qualitative analysis of semi-structured stakeholder meetings, as detailed below. We expanded on the adapted ERIC process by (a) incorporating user-centered design methods [31] to understand determinants and identify strategies related to the assessment and management of pHTN and (b) using the IRLM [32] as a conceptual and organizing framework. SAP meetings were held monthly for 7 months (April–October 2020), and once in January 2021, and lasted 1–2 h each. SAP members spent an average of 12 h in SAP meetings and related activities (e.g., surveys). Meetings occurred via Zoom videoconferencing platform [33], recorded with panelists’ permission, and analyzed by the research team. SAP members were compensated $150 per hour. For replication and generalizability purposes, we now discuss the method by outlining the stakeholder-engaged activities that resulted in the multilevel, multicomponent implementation strategy.

#### Step 1: Identifying barriers to adhering to the CPG for pHTN

SAP members were introduced to the project, meeting logistics, and project specifics by reviewing the study protocol [6]. The SAP then engaged in a semi-structured discussion of current practices in their respective clinics for measuring, diagnosing, and managing BP in children and adolescents, as well as identifying the barriers to adhering to the 2017 CPG for pHTN.

#### Steps 2 and 3: Understanding context and generating implementation strategies

Stakeholders participated in two user-centered design workshops [31]. First, they were asked to diagram and discuss their workflows for BP measurement, including (a) the pre-encounter vitals, (b) the clinician-patient encounter, and (c) the end-of-visit and follow-up plan. The research team prompted for barriers; communication channels between clinicians, staff, and families about BP results and treatment plans; and recommendations for strategies to overcome named barriers. Although this method was informed by the user-centered design literature [31], assessment and redesign of the workflow was recently suggested as an additional ERIC strategy [34]. Second, stakeholders were introduced to an EHR-integrated population health tool via a brief video and demonstration. They were then asked about how this tool may be useful for CPG adherence, additional clinical characteristics (e.g., BMI) needed for the tool to be useful, and potential ways such a tool could be integrated into routine practice.

#### Step 4: Defining implementation strategies

Following the generation of candidate strategies to improve pHTN CPG implementation, the SAP operationally defined each discrete strategy. This step was necessary for step 5 activities that involved linking strategies to identified barrier(s), and the later strategy specification in step 6.

Between the sessions comprising steps 4 and 5, the research team created a matrix of barriers by potential strategies the SAP identified through the activities of the first four steps (see Additional file 1). The goal was to elucidate the concordance of strategies with barriers and inform where SAP input was still needed.

#### Step 5: Review and confirm matrix of barriers and potential strategies

The SAP defined each barrier and indicated which strategies addressed each barrier. They were also encouraged to identify new barriers or new strategies to fill any gaps in the matrix. Before the next meeting, the research team consolidated and optimized the list of identified barriers by collapsing and pruning as conceptually and practically applicable.

#### Step 6: Specify the strategies in the matrix

Next, the SAP was shown the consolidated list of strategies and asked to specify the actor(s) (who does the strategy), action(s) (what the actors do), temporality (when the strategy was used), and dosage (the frequency and time of each use), per Proctor et al. [35].

### Rate strategies and determinants to inform prioritization and final selection

Next, panelists were invited to complete a survey (~30 min). First, they rated each determinant: –2 (strong, negative impact on implementation; i.e., strong barrier), –1, 0 (neutral impact), +1, +2 (strong, positive impact on implementation; i.e., strong facilitator) [36]. Second, panelists completed ratings of each strategy’s perceived effectiveness, feasibility, and priority for their community health center on a scale from 1 (low) to 4 (high) per the ERIC protocol [30].

**Fig. 2:**
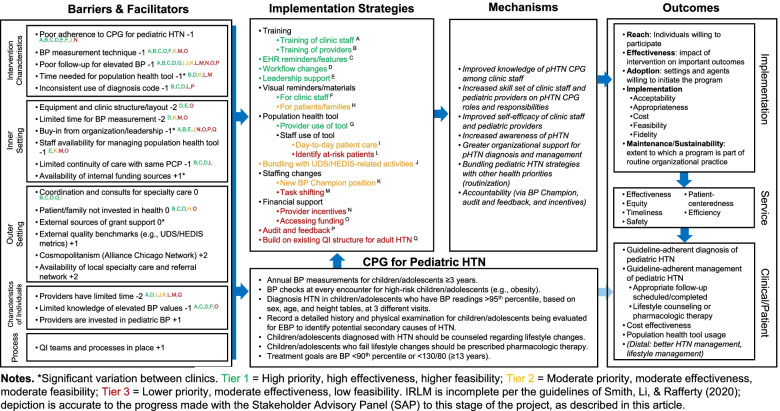
Implementation Research Logic Model (IRLM) for OpTIMISe strategy selection and prioritization

Using the strategy ratings, the research team used a three-tier approach to prioritization, which largely reflected a natural division in the ratings (described below in the “Data analysis” section). To facilitate the process of prioritization with the SAP, the research team populated the determinants and strategies sections of the IRLM (Fig. 2) and used the matrix of determinants and strategies (created in accordance with ERIC steps 3 and 4 and following step 5) to indicate the relationships between them using superscripts (e.g., the population health tool strategy addresses the determinants of poor follow-up for elevated BP and coordination and consults for specialty care)—a recommended step in using the IRLM [29, 32]. This step helped the SAP assess the degree of coverage the proposed strategies provided for the prominent barriers (step 7).

#### Step 7: Build consensus on the prioritization of strategy package using the IRLM

The IRLM was presented to the SAP with ratings, superscripts, and proposed prioritization of strategies as described above using the three-tier approach. The SAP was instructed to examine the coverage of the primary barriers with the proposed strategies in tier 1. Deficiencies in coverage of barriers in the tier 1 strategies resulted in elevating strategies from tier 2 to tier 1 and adding two new strategies that had not been previously discussed. We repeated the step of specifying these new strategies as done in step 6, but the survey ratings were not repeated as their prioritization (i.e., tier assignment) was clear from discussion during the session. Finally, because the identified determinants to this point were largely barriers, the SAP was asked to identify facilitators; seven were identified and rated through group consensus.

#### Step 8: Obtain stakeholder buy-in and feedback on project proposal

Approximately 3 months after the meeting to complete step 7, the SAP was convened to reflect on and review the strategies being proposed in a grant application to support an implementation trial (described in the “Discussion” section). The SAP was shown the final IRLM (including mechanisms and outcomes) and the supporting text describing the processes of the SAP, the study approach, and their proposed involvement in the project should it be awarded.

#### Complementary activities

In addition to the SAP meetings, the research team elicited input from caregivers of children with, or at risk for, pHTN and from clinic staff (i.e., nurses, medical assistants) based on the evolving strategy plan and identified barriers. Caregivers identified many similar determinants of pHTN diagnosis and treatment as the SAP (e.g., concern about elevated BP in their children). Clinic staff confirmed the feasibility and acceptability of all strategies presented to them and provided important details to increase the likelihood of implementation success (e.g., integrating follow-up or booster trainings into pre-existing staff activities, such as “lunch and learn” sessions and team huddles). See Additional file 2 for the full report of the methods and results of these complementary stakeholder activities.

### Data analysis

The transcripts from steps 1–4 were analyzed using Rapid Turnaround Qualitative Analysis [37, 38]. Two members of the research team completed two 4-h trainings in Rapid Turnaround Qualitative Analysis for implementation research (conducted by ABH). The first two SAP sessions were double-coded and results were compared and discussed before sessions 3–4 were coded by a single rater. Coding was undertaken to identify determinants and corresponding strategies, in accordance with the five domains of the Consolidated Framework for Implementation Research (CFIR) [39]. Coding was also informed by the recommendations for implementing health information technology (HIT) tools [40].

Descriptive quantitative analyses of the survey, including means, ranges, and relative rankings, were used to rate determinants and prioritize strategies. First, the mean ratings of the determinants were rounded to the nearest whole integer (−2, −1, 0, +1, +2) and determinants were characterized as barriers (mean ratings <0) and facilitators (mean ratings >0) [36]. Second, the mean ratings of the strategies’ feasibility, effectiveness, and prioritization were compiled, and strategies were grouped into three tiers. Tier 1 included strategies that were rated to be highest priority, high effectiveness, and higher feasibility. Tier 2 included strategies that were rated to be moderate priority, moderate effectiveness, and moderate feasibility. Tier 3 included strategies that were rated to be lower priority, moderate effectiveness, and lowest feasibility. Determinations were made for each strategy relative to the others as no clear thresholds or cut points exist for such ratings.

## Results

### Selection of the implementation strategy

#### SAP meetings and ratings

The primary goal of the semi-structured SAP meetings was to engage stakeholders in identifying the primary determinants and strategies for implementing the 2017 CPG for pHTN into primary care practices in community health centers. Across activities, 14 determinants were identified. Stakeholder ratings of the strength of each determinant resulted in mean scores ranging from −1.71 (strong barrier) to +.14 (moderate facilitator), with a mean overall score of −.95 (see Table 1 for determinant and their ratings used in the IRLM). This overall mean score is consistent with the sessions being designed to elicit barriers more so than facilitators. Thus, the final SAP meeting included asking panelists to identify facilitators and rate their strengths. Six new facilitators were identified; scores ranged from +1 to +2, with an average score of +1.33 (moderately strong).

**Table 1:** Stakeholder ratings of determinants used in the logic model

Determinant	*Mean (SD)*	*Range*
Poor adherence to CPG for pediatric HTN	−1.10 (0.73)	−2–0
BP measurement technique	−1.30 (0.76)	−2–0
Equipment and clinic structure/layout limitations	−1.50 (0.5)	−2 to −1
MAs/nurses not aware of elevated BP values	−0.09 (0.69)	−2–0
Limited time for BP repeats and during patient encounters	−1.70 (0.49)	−2 to −1
Poor patient follow-up for repeat visit: frequent no-shows and cancelations, provider is responsible for ensuring patients follow up	−1.30 (0.76)	−1–0
Limited continuity of care for (some) patients	−0.60 (0.53)	−1–0
Inconsistent use of elevated BP/pediatric HTN diagnosis (e.g., on problem list)	−0.60 (0.53)	−1–0
Coordination and consults for EBP	−0.10 (0.90)	−1–1
Patient/family not invested in health	−0.10 (0.69)	−1–1
Need for buy-in from the clinic and organization to prioritize BP training and initiatives	−0.40 (1.27)	−2–2
Time it takes to setup Population Health panel	−0.90 (1.07)	−2–1
Person responsible for managing a Population Health panel	−1.30 (0.95)	−2–0
Pediatric clinicians have limited time to add a new task to workflow; population health tools may not be practical for day-to-day patient care	−1.60 (0.53)	−2 to −1
Overall	−.95 (.52)	−1.71–.14

#### Selected implementation strategies

Across the SAP meetings and interviews with clinic staff, 18 discrete strategies were identified, defined, and specified (i.e., actors, actions, temporality, and doses) (see Table 2). While the identified strategies primarily targeted determinants in the CFIR domains of intervention characteristics (e.g., elements of the CPG), inner setting (e.g., equipment, leadership support), and implementer characteristics (e.g., staff awareness of BP value interpretation), there were also strategies addressing barriers in the outer setting and process domains. The strategies primarily involved the practice’s pediatric clinician or healthcare staff as the actors, and actions occurred most often during point-of-care interactions, with the exception of population health strategies.

**Table 2:** Discrete strategies and specifications identified by stakeholders across interviews and workshops

Strategy category	Discrete strategy	Actor(s)	Action(s)	Temporality	Dosage
**Training**	Asynchronous training of MA/nurses	Trainers: video (School of Medicine, available materials with pediatric standardized patient) Trainees: MAs/nurses	• Training in BP measurement techniques and context (refreshers) • Training in EHR strategies for EBP	• New hires • Transfers/inter-departmental changes to pediatrics (within 1 week of starting)	• 20–30 min
	Synchronous training of MA/nurses	Trainers: BP champion(s), MA trainer or lead MA Trainees: MAs/nurses	• In vivo BP measurement technique and context training • Training in EHR strategies for EBP • BP measurement spot-checks	• Immediately following asynchronous training (above) • Spot-checks: ongoing	• 15–20 min (total) • Spot-checks: quarterly, 5–10 min
	Training of MA/nurses in manual BP reading	Trainers: BP champion(s), Video (School of Medicine, available materials with pediatric standardized patient) Trainees: MAs/nurses	• Training in manual BP measurement techniques • Manual BP measurement spot-checks	• New hires • Transfers/inter-departmental changes to pediatrics (within 1 week of starting) • Spot-checks: ongoing	• 20–30 min
	Asynchronous training of pediatric clinicians	Trainer: video Trainees: pediatric clinicians	• Training in BP measurement techniques and context (particularly for pediatric practices) • How to address EBP, etc. • Training in EHR strategies	• Annually	• One training for diagnosis • One training for treatment/management (~15 min each)
	Synchronous training of pediatric clinicians	Trainer: specialist (e.g., pediatric nephrologist) Trainees: pediatric clinicians	• Psychoeducation (grand rounds, in-service, counseling strategies, case presentations)	• Annually	• 50–60 min
**Audit and feedback**	Feedback reports	• Data team • Alliance-generated report • Targets: pediatric clinicians, (support staff)	• Dashboard report • Practice-level comparisons, • Individual clinician performance (combined with annual review)	• Report generation and review: as needed (recommended: monthly to quarterly, combined with other data reports/reviews) • To providers: 6 months (combined with incentive structure)	• ~10 min per meeting
**Workflow changes**	Workflow changes	Operations director (to MAs)	• Specification and dissemination of new workflow	• Within 1 month of implementation launch	• 1–2 h
**Staffing changes**	Develop new position	• Operations director/practice manager • Residency/intern manager	• Describe the position and hire; develop a business plan to justify position	• Within 1 month of implementation launch	• 6–8 h
	Shift tasks among existing positions	• Operations director/practice manager • Residency/intern manager	• Specify new expectations (population health tool) • Relieve other duties and reassign as needed	• Within 1 month of the launch of the population health tool	• 1–2 h
**Visual reminders/materials (non-digital)**	Visual reminders for staff	• Operations director/practice manager • Marketing team	• Make materials available and accessible	• Within 1 month of implementation launch • Refresh as needed	• 2–5 min per patient, as needed (to use materials) • May require time to create materials
	Materials for patients/families	• Operations director (for workflow change) • MAs (sending messages)	• Send email or snail mail to indicated patients/families	• Within 1 month of implementation launch • Refresh as needed	• 1–2 h/week
**HIT solutions/features**	EHR reminders and features	• Alliance, individual health center’s EHR department • Target: pediatric clinicians, support staff	• Programming EHR (order sets, clinical decision tree quick-link)	• Within 1 month of implementation launch, use with every patient as necessary	• 1–2 min/patient
**Population builder (population health tool)**	Identify at-risk patients/populations	• BP champion(s) • Trainees • Data team • Case managers	• Run population queries and review • Flag at-risk patients (scheduled or need follow-up)	• Weekly to monthly	• 5–20 min/week (highly variable)
	Patient care huddles	• Care team (pediatric clinicians and support staff, case managers)	• Meeting to review results of population health tool query	• Daily	• 5–10 min, 1–2 times/day
**Leadership support**	Engaging leadership	• Pediatric clinicians • Quality Improvement team • External actors from relevant interest groups (AAP, AllianceChicago)	• Meetings • Materials to make the case • Prioritization within strategic plan/quality improvement plan	• Highly variable per CHC (more effort up front, with ongoing time commitment)	• Variable, dependent on the current stage of change
**Financial support**	Provider incentives	• Leadership: COO, CEO, CFO • HRSA, • UDS measures • Insurance companies	• Integrate within existing pediatric provider incentive structure plan and financial model	• 6 months (performance review schedule)	• Requires 2–3 h up front, minimal time once integrated
	Accessing funding (positions, equipment)	• Leadership: COO, CEO, CFO	• Add/integrate into yearly budget • Clinic space: work with facilities	• Ongoing (e.g., replace broken equipment, as needs arise)	• 30–60 min for budget planning

*Notes*. Actor indicates “Who does this?”; Action(s) indicate “what do the actors do?”; Temporality specifies “When was the strategy used?”; and Dosage refers to frequency of use and time involved in each use. We do not include “action target,” “implementation outcome,” or “justification”, which are elements of the Proctor et al. (2013) suggestions for specifying strategies. This is because some of these appear elsewhere in the IRLM. For example, superscripts in Fig. 2 indicate linkages between strategy and determinant (which is often part of “action target”), and potential mechanisms are described as well, which are part of both “action target” and “justification”; implementation outcomes are also included in the IRLM

### Using the IRLM

The completed IRLM appears in Fig. 2. One of the final steps needed to convert the results of the adapted ERIC process and other activities to the IRLM was to determine the tier of each strategy based on stakeholder reporting priority, effectiveness, and feasibility, as well as alignment with peer-reviewed evidence of their effectiveness [40, 41].

Concerning the ratings, scores (Table 3) ranged from 1 to 4 for priority (*M*=2.97; *SD*=0.89), 2 to 4 for effectiveness (*M*=3.23; *SD*=0.68), and 1 to 4 for feasibility (*M*=2.82; *SD*=0.98). Determinant-strategy links are noted with capitalized superscript letters, and the tiers are reflected by text color (i.e., green: tier 1; yellow: tier 2, red: tier 3), grouped according to ERIC strategy category [30].

**Table 3:** Stakeholder ratings of strategy effectiveness, feasibility, and priority

Strategy	Feasibility		Effectiveness		Prioritization	
	*M (SD)*	*Range*	*M (SD)*	*Range*	*M (SD)*	*Range*
Asynchronous training of MA/nurses	3.57 (0.53)	3–4	3.57 (0.53)	3–4	3.29 (0.76)	2–4
Synchronous training of MA/nurses	2.86 (0.69)	2–4	3.57 (0.53)	3–4	3.14 (0.69)	2–4
Training of MA/nurses in manual BP reading	2.86 (0.69)	2–4	3.57 (0.53)	3–4	3.43 (0.79)	2–4
Asynchronous training of pediatric clinicians	3.71 (0.49)	3–4	3.57 (0.79)	2–4	3.29 (0.76)	2–4
Synchronous training of pediatric clinicians	3.00 (1.15)	1–4	3.14 (0.69)	2–4	3.14 (0.69)	2–4
Feedback reports	2.86 (0.69)	2–4	3.00 (0.58)	2–4	2.86 (0.69)	2–4
Workflow changes	2.86 (0.69)	2–4	3.14 (0.90)	2–4	3.43 (0.79)	2–4
Develop a new position	1.86 (1.07)	1–4	3.14 (0.69)	2–4	2.29 (0.95)	1–4
shift tasks among existing positions	2.43 (0.79)	2–4	3.00 (0.00)	3–3	2.57 (0.53)	2–3
Visual reminders for staff	3.71 (0.49)	3–4	3.43 (0.79)	2–4	3.71 (0.49)	3–4
Materials for patients/families	3.29 (1.11)	1–4	2.86 (0.38)	2–3	2.71 (0.95)	1–4
EHR reminders and features	3.14 (0.69)	2–4	3.43 (0.53)	3–4	3.43 (0.79)	2–4
Identify at-risk patients/populations	2.71 (0.76)	2–4	3.00 (0.58)	2–4	2.86 (0.69)	2–4
Patient care huddles	3.29 (0.95)	2–4	3.29 (0.76)	2–4	2.86 (1.21)	1–4
Engaging leadership	2.43 (0.79)	1–3	3.14 (0.69)	2–4	2.71 (0.95)	1–4
Pediatric clinician incentives	1.57 (1.13)	1–4	3.00 (1.00)	2–4	2.29 (1.11)	1–4
Accessing funding (positions, equipment)	1.86 (0.69)	1–3	3.00 (1.00)	2–4	2.57 (1.13)	1–4
Overall	2.82 (0.98)	1–4	3.23 (0.68)	2–4	2.97 (0.89)	1–4

We then looked to the literature and verified that all of the implementation strategies selected by the SAP, particularly those in tier 1 (i.e., those with the highest priority, effectiveness, and feasibility), were supported as effective strategies to promote CPG adherence generally [28] and for hypertension in particular [42]. For example, training and education of pediatric clinicians and staff is commonly used through distribution of educational materials, group meetings and supervision, and formal training seminars [43, 44]. Our SAP members were also interested in using novel HIT strategies (e.g., EHR reminders, population health tools), which have proven successful in prior studies in this clinic network (e.g., [45]). To support these strategies, we closely followed the recommendations for implementing e-health strategies set forth by Ross et al. [40], which included considering compatibility with existing systems and practices, planning for implementation as well as ongoing monitoring and evaluation, training, and education for those involved, and amassing support from key stakeholders and leaders. Finally, for each of these strategies, we worked closely with the SAP to tailor the implementation strategies for the specific context of the primary care practices in community health clinics, as recommended by Fisher et al. [28].

After verifying the contents with stakeholders for accuracy and completeness, the IRLM was used to finalize the proposed multilevel, multicomponent implementation strategy for adherence to the CPG for pHTN. The two primary factors determining placement in the lower tiers, compared to tier 1, were cost and divergence from current practice, which reflects feasibility concerns. This consideration resulted in many strategies being understood to be minor-moderate adjustments to current practices rather than major changes. This aligns with the premise of this project that aims to improve adherence to a CPG that has many of its elements already as standard of care, but not in the precise manner specified by the CPG.

## Discussion

Given the omnipresent research-to-practice gap, many models and frameworks have been proposed to narrow that gap and improve the speed of translation [46]. Several approaches (e.g., community-engaged research, user-centered design) emphasize the importance of stakeholders in the selection and tailoring of implementation strategies [3]. Stakeholders provide critical insights about implementation factors, such as intimate knowledge of workflows, organizational infrastructure, culture, available resources, and other challenges that could inhibit successful implementation. This article presents a generalizable method that expanded the adapted ERIC protocol to meaningfully engage stakeholders in the identification and prioritization of implementation strategies. This method was exemplified through a case example that worked closely with stakeholders to select implementation strategies to support adherence to the CPG for pHTN [47].

We augmented the adapted ERIC protocol used in previous research [29, 30] in two unique ways. First, two user-centered design workshops complemented the information elicited via the semi-structured SAP sessions. Specifically, when stakeholders diagrammed their workflow and interacted with the population health tool, existing determinants and implementation strategies were confirmed, and new ones were elicited. Other user-centered design activities could be used to complement the ERIC protocol. For example, given the restricted range in scores for determinant and strategy ratings obtained via our survey, as well as their conceptual overlap, card sorting may be a useful activity for both priority ranking strategies and categorizing similar determinants and strategies, similar to what was done in the concept mapping stage of ERIC [1]. Our approach has the potential to increase the likelihood of success of the strategies once enacted due to deep stakeholder engagement in the selection process.

A major strength of this method is the sequential, iterative meetings allowed us to first identify and then adapt the determinants and implementation strategies to focus on in subsequent stakeholder meetings. For example, in early meetings, we noticed most of the determinants identified by stakeholders were barriers (cf., facilitators). After analysis of early SAP meetings revealed this pattern, we were able to specifically ask stakeholders to identify facilitators in a subsequent meeting rather than relying on existing literature or other sources for this information, or it being absent. After the SAP meetings, we confirmed the acceptability and feasibility of selected strategies with additional stakeholders who would be involved or impacted by them (i.e., caregivers, clinic staff). This approach differs from typical data collection procedures that are often a fixed endeavor and, even if data collection is prospective, protocols and assessments are less adaptable even as relevant findings accrue.

Second, using the IRLM as an organizing framework and visual aid allowed us to connect determinants to strategies with desired implementation outcomes and prioritize strategies. Connecting determinants to strategies is not only recommended when specifying each discrete strategy [35], but it was also found to be useful in evaluating whether the proposed strategies appropriately addressed the barriers identified—a critical challenge when designing a multilevel, multicomponent implementation strategy for a complex problem like pHTN CPG adherence, and in the field more generally [48, 49]. Additionally, the IRLM enabled pruning and prioritizing strategies using the tiering process as shown in Fig. 2. One of our main goals of this project was to define feasible and effective implementation strategies that could be empirically optimized (e.g., improved over time with multiple iterations) in a subsequent implementation trial. Thus, rather than simply selecting a single multicomponent strategy, we placed discrete strategies in tiers as a way to facilitate the desired optimization approach (described next). Finally, using the IRLM as a final organizing tool led to the SAP generating two additional strategies after viewing the alignment of the strategies with the primary barriers.

### Preparation for a subsequent implementation trial

The adapted and expanded ERIC process described here was partially designed to obtain preliminary data for a randomized optimization trial design, the Roll-Out Implementation Optimization (ROIO) design [50]. This design differs from traditional roll-out designs in that the explicit goal is an empirically driven optimization of implementation strategy effectiveness between clusters. The strategy tiering approach was instrumental to preparing for a ROIO trial as the tier 1 strategy package will serve as the first package tested in cluster 1, and the strategies from tier 2 (and tier 3 if needed) will serve as an already-vetted menu of options from which to choose in subsequent clusters. The IRLM furthered this goal because the conceptual connections between determinants, strategies, and outcomes are central to the deliberation process for choosing additional/new strategies, or removing ineffective strategies, in a way that is likely to lead to improvements in specific outcomes. While the adapted and expanded ERIC protocol used for this study was well aligned with the needs of the ROIO design, other optimization designs, adaptive designs, and comparative implementation trial designs [51] could all benefit from such a process in the planning phase. Regardless of the study design, the method described in this article can be used for identification, specification, and prioritization of strategies given that many implementation efforts will require multiple strategies that require buy-in from implementation partners and consideration of feasibility.

### Limitations and other considerations

The current project should be considered in light of certain limitations. First, while some stakeholders additionally held clinical leadership and quality improvement positions, the case example did not include executive leadership members (e.g., Chief Medical Officer, Chief Operations Officers), policymakers, or payors, whose perspectives in determining the optimal strategy will be critical for uptake and sustainment of any organizational change. For projects considering using this method, it will be important to include stakeholders across organization levels as is both justifiable and feasible. Furthermore, the SAP members for the exemplar study were passionate about pediatric cardiovascular health and quality improvement, which may be an important factor to consider when forming stakeholder panels in future studies using this method. Second, while not a limitation specific to this project, the short-term mechanism that funded this work allowed for deep stakeholder engagement, a rigorous process spanning nearly a year. Such a timeline for implementation strategy development may not be feasible for all projects wanting to use this method, but would be possible using smaller funding mechanisms (e.g., R03, R21) [52]. Given how critical stakeholder engagement is to the success of implementation research, our field needs to rethink the small amount of time and financial support typically allocated for partnership development and stakeholder engagement prior to implementation trials. Additionally, it is worth noting that the case example’s project length was likely due to the complexity of the guidelines and multilevel, multicomponent strategy package, suggesting the method could be used on a smaller scale and in a shorter time period depending on project scope. Our team, led by JDS, AJK, and AJC, recently completed an abbreviated version comprising individual interviews (approximately 60 minutes) and three 2-hour group meetings of the method described in this paper to similar success for a project focused on implementation of an evidence-based screening and preventive intervention system for toddler social-emotional wellbeing in community-based pediatric practices.

## Conclusion

This article presents a generalizable method to meaningfully engage stakeholders in the selection and specification of a multilevel, multicomponent implementation strategy. We illustrated the use of this method in the context of a project focused on pHTN CPG implementation. The procedures used for this project to select an implementation strategy, which involved an adapted ERIC protocol augmented with user-centered design methods and the IRLM, provide a generalizable approach that can be applied to other implementation challenges to improve the likelihood of strategy adoption, effectiveness, and sustainment.

## Supplementary Information

**Additional file 1.** Determinants—Strategies Matrix.

**Additional file 2.** Complementary Activities Resulting from Additional Stakeholder Involvement.

## Data Availability

Data and materials are available upon request to the corresponding author.

## Abbreviations

BP: Blood pressure
CFIR: Consolidated Framework for Implementation Research
CPG: Clinical practice guideline
EHR: Electronic health record
ERIC: Expert Recommendations for Implementing Change
IRLM: Implementation Research Logic Model
pHTN: Pediatric hypertension
SAP: Stakeholder Advisory Panel
HIT: Health-information technology
