# Supplementary material for: A stakeholder-driven method for selecting implementation strategies: a case example of pediatric hypertension clinical practice guideline implementation
Source: Implement Sci Commun. 2022 Mar 7;3:25. doi: 10.1186/s43058-022-00276-4 (PMC8900435; doi:10.1186/s43058-022-00276-4)
Supplement: Supplementary file 2 — Additional file 2. Complementary Activities Resulting from Additional Stakeholder Involvement. [file 43058_2022_276_MOESM2_ESM.docx]

**Additional File 2**

**Complementary Activities Resulting from Additional Stakeholder Involvement**

**Methods**

**Caregivers of children at risk for pediatric hypertension.** At the recommendation of the SAP members and our Scientific Advisory Board, we convened a meeting of caregivers (n=6) of children with, or at-risk for, pHTN.

**Clinic Staff.** Nurses and medical assistants (n=6) who specialized in pediatrics or family medicine and worked with children and their families in community health center primary care practices were invited to participate in a one-time, one-on-one interview to understand their role in the diagnosis and management of pHTN, discuss the initial implementation strategy package as identified by the SAP.

**Procedures**

**Perspectives of caregivers of children at risk for or diagnosed with pHTN.** We convened a Stakeholder Academic Resource Panel (ShARP) (37) comprising caregivers of children with or at-risk of pHTN. ShARPs aim to promote community-engaged research. They are a service of the Center for Community Health at Northwestern, who recruits, facilitates, and provides compensation of ShARP participants. Caregivers were asked about their personal, family, and child’s history with hypertension, including their understanding of hypertension and pHTN, the importance of having blood pressure measured and discussed at each doctor’s visit, and perceptions regarding treatment and treatment adherence for hypertension. The ShARP took place in October 2020, lasted 90 minutes, and was recorded in Zoom (33). The discussion was analyzed by the Center for Community Health team, who then provided an executive summary to the research team.

**Feedback from clinic staff on staff-focused strategies.** After the Tier 1 strategies were identified, we sought input on the acceptability, feasibility, and specification of the strategies that would be undertaken by or would impact clinic staff. Nurses and medical assistants were identified by a member of the research team who is on the leadership of AllianceChicago (NM) and invited to participate in a one-time, 30-minute, semi-structured interview. Interviews took place between December 2020 and January 2021, were recorded in Zoom (33) with interviewees’ permission, and analyzed by the research team. Clinic staff were compensated $50 for the 30-minute interview.

**Data Analysis**

The feedback from the clinic staff interviews were analyzed using Rapid Turnaround Qualitative Analysis (37, 38). Two members of the research team completed two 4-hour trainings in Rapid Turnaround Qualitative Analysis for implementation research (conducted by ABH). The six clinic staff interviews were single-coded and responses discussed and consolidated. Coding was undertaken to identify determinants and strategies to address barriers, in accordance with the five domains of the Consolidated Framework for Implementation Research (CFIR) (39). Coding was also informed by the recommendations for implementing HIT tools (40).

**Results**

**Caregiver panel.** The executive summary of the ShARP indicated that the caregivers identified many similar determinants of pHTN diagnosis and treatment as the SAP: worries about elevated BP in their children and being invested in its prevention or treatment. Caregivers also noted the following barriers: 1) difficulty changing family behaviors (e.g., family diet), 2) limited time during doctor’s visits dedicated to measuring and discussing BP, and 3) needing to prioritize other health concerns (e.g., attend to other medical concerns vs. attending follow-up visits for BP). Caregivers also indicated high interest in provision of additional information from pediatric providers regarding the importance of children’s BP measurement and treatment.

**Clinic staff interviews.** Most determinants raised by clinic staff coincided with the barriers rated strongest by pediatric providers, such as limited time for BP measurements and lack of proper equipment. Clinic staff also mentioned parent understanding of the importance and consequences of elevated BP in children being a determinant. Specific clinic staff training needs involved manual BP measurement, familiarity with EHR functionality, educating families on the consequences of elevated BP, and adhering to the required follow-up appointments (i.e., different timeline for those with elevated BP vs. those meeting criteria for pHTN). Further, they agreed the training-based strategies specific to their needs were acceptable and feasible. They confirmed that spot-checking BP measurement skills, particularly for manual measurement, every 3-6 months would appropriately assess their knowledge and skills. They added that clinic staff follow-up or booster trainings could occur in pre-existing activities (e.g., “lunch and learn” sessions, team huddles). They provided additional details regarding the BP champion: either a part- or full-time position depending on size of the clinic, time to conduct trainings and booster sessions, and being a general “go-to” BP provider. To help educate families, clinic staff mentioned the utility of handouts for caregivers and children regarding elevated BP and pHTN.
